# Mapping Antimicrobial Resistance in *Staphylococcus epidermidis* Isolates from Subclinical Mastitis in Danish Dairy Cows

**DOI:** 10.3390/antibiotics14010067

**Published:** 2025-01-10

**Authors:** Desiree Corvera Kløve, Mikael Lenz Strube, Peter M. H. Heegaard, Lærke Boye Astrup

**Affiliations:** 1 Department of Health Technology, Technical University of Denmark, Kemitorvet, 2800 Kongens Lyngby, Denmark; 2 Department of Biotechnology and Biomedicine, Technical University of Denmark, Søltofts Plads, 2800 Kongens Lyngby, Denmark; 3 SEGES Innovation P/S, Agro Food Park, 8200 Aarhus, Denmark

**Keywords:** antimicrobial resistance, antimicrobial resistance genes, antimicrobial susceptibility testing, bovine mastitis, genetic diversity, minimum inhibitory concentration, non-aureus staphylococci, *Staphylococcus epidermidis*, whole-genome sequencing

## Abstract

**Background/Objectives:** Although *Staphylococcus epidermidis* is a key cause of subclinical mastitis in Danish dairy cows, its sensitivity to antimicrobials remains unexplored. Here, we analyzed sixty *S. epidermidis* isolates derived from 42 dairy cows across six conventional dairy herds in Denmark. **Methods:** Phenotypic resistance was measured by antimicrobial susceptibility testing and minimum inhibitory concentration (MIC) analysis, and genotypic resistance was examined through whole-genome sequencing and identification of antimicrobial resistance genes (ARGs). Correspondence between phenotypic and genotypic resistance was then evaluated by Cohen’s kappa statistics. Furthermore, the presence of plasmid replicon genes and the strain diversity among the *S. epidermidis* isolates was investigated to associate these findings with the observed AMR patterns. **Results:** Results showed that 30/60 isolates (50.0%) were resistant to penicillin phenotypically, while 35/60 (58.3%) were positive for a corresponding *blaZ* gene (*κ* = 0.83, *p* < 0.01). A *fosB* gene, encoding fosfomycin resistance, was detected in all 60/60 isolates (100.0%), but fosfomycin resistance was not analyzed phenotypically. Based on MIC analysis, 3/60 isolates (5.0%) were multi-drug resistant, showing resistance towards penicillin, erythromycin, and tetracycline. However, in 11/60 genomes (18.3%), ARGs encoding resistance towards ≥3 antimicrobial classes (e.g., beta-lactams, phosphonic acid, tetracyclines, aminoglycosides, macrolides, lincosamides, and fusidane) were detected. Eleven different ARGs were detected among the 60 isolates in total. No methicillin-resistant *Staphylococcus epidermidis* (MRSE) were recorded. Results further showed that each herd had one primary sequence type (ST) and resistance profile associated with it, and plasmid-mediated horizontal gene transfer of ARGs was indicated This study underscores the importance of routine resistance surveillance and species-specific diagnoses to improve treatment outcomes and ensure prudent use of antimicrobials.

## 1. Introduction

Antimicrobials are used for treating infectious diseases in both humans and animals. However, their use drives the emergence and spread of antimicrobial resistance (AMR), which poses a growing threat to global health [1]. To address this threat, the prudent use of antimicrobials is promoted across both the healthcare and agricultural sectors.

The production of milk and dairy products is a substantial part of global food production [2]. Mastitis is the most common disease in dairy cows and is defined as an inflammatory response in the udder often due to intramammary infection caused by bacteria [3,4]. Furthermore, treatment and control of mastitis is the leading cause of antimicrobials usage in dairy cows, although treatment practices vary between countries and herds [3,5,6]. In Denmark, penicillins are primarily used for mastitis treatment in dairy cows [5,7].

Non-aureus staphylococci (NAS) are frequently associated with subclinical mastitis (SCM) (i.e., inflammation of the udder characterized by the absence of visible signs, hence, SCM is detected by an increased somatic cell count (SCC) in the milk), and, to some extent, clinical mastitis (CM) (i.e., inflammation of the udder characterized by visible abnormalities in milk, udder, or cow) [4,8,9]. *Staphylococcus epidermidis* is an example of an NAS commonly found in SCM [8]. A recent national large-scale study investigated the prevalence of bacteria associated with SCM in Danish dairy cows and found *S. epidermidis* as one of the most abundant NAS [10]. Although *S. epidermidis* is one of the most common bacteria associated with SCM in Denmark and internationally [11], knowledge of its resistance prevalence and strain diversity is sparse.

Surveilling resistance is a crucial tool in fighting AMR as it monitors resistance patterns, identifies emerging trends, and may guide effective treatment [1]. Studies on AMR prevalences in mastitis pathogens are often based on the detection of phenotypic resistance and minimum inhibitory concentration (MIC) analysis [8,12,13], but studies further investigating genotypic resistance by identifying antimicrobial resistance genes (ARGs) are increasing [8,14,15]. Irrespective of whether genotypic resistance is added to the methodology or not, resistance surveillance is important across all sectors, as (resistant) pathogens may spread between different hosts. However, studies on the resistance surveillance of mastitis pathogens in Danish dairy cows are very limited. Therefore, this study was initiated with the aim of mapping the AMR prevalence in bovine *S. epidermidis*. For this, phenotypic and genotypic resistance were investigated by MIC analysis and whole-genome sequencing to identify ARGs, respectively. The whole-genomes of the isolates were further analyzed to study the strain diversity of *S. epidermidis* and the presence of plasmid replicon genes to investigate the possible association of these factors with the AMR patterns observed.

## 2. Results

This study is based on 60 *S. epidermidis* isolates derived from SCM in Danish dairy cows (Table 1). The isolates were selected from a bacterial strain collection previously collected as part of a large prevalence study during 2019–2021 in Denmark (data to be published). For metadata and further details regarding their selection, we refer to Section 4.1, Tables S1 and S2.

### 2.1. Phenotypic Antimicrobial Resistance

The 60 *S. epidermidis* isolates were tested for their susceptibility towards 14 antimicrobial agents (Table 2). For 7/14 agents (cefoxitin, ciprofloxacin, erythromycin, gentamicin, penicillin, tetracycline, and trimethoprim), epidemiological cut-off values (ECOFFs) from EUCAST were available and used in MIC analysis to determine the occurrence of phenotypic resistance (non-wild type isolates with detectable resistance mechanisms phenotypically) [16]. The highest phenotypic resistance level was for penicillin found in 30/60 isolates (50.0%), followed by tetracycline found in 8/60 isolates (13.3%), and erythromycin found in 5/60 isolates (8.3%). All 60 *S. epidermidis* isolates were fully sensitive to ciprofloxacin, gentamicin, and trimethoprim. Furthermore, the isolates were all sensitive to cefoxitin, thus the presence of methicillin-resistant *Staphylococcus epidermidis* (MRSE) isolates was not indicated (Table 2). For the remaining seven agents, where no ECOFFs are available (chloramphenicol, florfenicol, spectinomycin, streptomycin, sulphamethoxazole, tiamulin and trimethoprim + sulphamethoxazole (TMP + SMX)), a bimodular MIC distribution was observed for, respectively, sulphamethoxazole and tiamulin, indicating the presence of a non-wildtype population (Table 2). For chloramphenicol, florfenicol, spectinomycin, and TMP + SMX, a narrow unimodal MIC distribution was observed indicating only a wild-type population. For streptomycin, a wide unimodal MIC distribution was seen, with the entire test range being represented (Table 2). Finally, 3/60 isolates (5.0%) showed resistance towards three agents belonging to three different antimicrobial classes (penicillin, i.e., beta-lactams, tetracycline, i.e., tetracyclines, and erythromycin, i.e., macrolides), hence they were deemed multi-drug resistant (MDR) [17]. Furthermore, these isolates shared herd of origin (herd 6).

### 2.2. Genotypic Antimicrobial Resistance

The 60 *S. epidermidis* isolates were whole-genome sequenced to investigate the presence of ARGs and to explore the correspondence between phenotypic and genotypic resistance for the isolates. The latter was assessed using Cohen’s kappa statistics. However, Cohen’s kappa statistics were applied only for the 7/14 antimicrobials where an ECOFF was available (Table 2).

Eleven different ARGs were identified among the 60 *S. epidermidis* genomes (Table 3). The most prevalent ARG was *fosB,* encoding fosfomycin resistance, detected in 60/60 isolates (100.0%). However, fosfomycin resistance was not tested in MIC analysis (Table 2). The second most prevalent ARG was *blaZ,* encoding penicillin resistance, found in 35/60 isolates (58.3%). Of note, only 30/60 (50.0%) isolates showed a penicillin resistant phenotype in the MIC analysis (Table 2). However, according to Cohen’s kappa statistics, there was an almost perfect agreement between genotypic and phenotypic resistance to penicillin (*κ* = 0.83, *p* < 0.01) (Table 4). The remaining ARGs identified among the genomes were present in less than 10% and were involved in resistance to aminoglycosides, lincosamides, fusidane, macrolides, and tetracyclines (Table 3). For erythromycin (belonging to the class of macrolides), 5/60 isolates (8.3%) were measured phenotypically resistant in MIC analysis (Table 2). Accordingly, 5/60 isolates (8.3%) were carrying two corresponding ARGs; *msr(A)* and *mph(C)*, and Cohen’s kappa statistics demonstrated an almost perfect agreement between phenotypic and genotypic resistance (*κ* = 1, *p* = 0.00) (Table 4). For tetracycline, a *tet(K)* ARG was detected in 5/60 isolates (8.3%), while 8/60 isolates (13.3%) showed a tetracycline resistant phenotype in MIC analysis. According to Cohen’s kappa statistics, a substantial agreement was found between phenotypic and genotypic tetracycline resistance (*κ* = 0.74, *p* < 0.01) (Table 4). Cohen’s kappa was not calculated for cefoxitin, ciprofloxacin, gentamicin, and trimethoprim as the 60 *S. epidermidis* isolates tested were fully susceptible in MIC analysis, while furthermore having no ARGs encoding resistance to these antimicrobials detected in their genomes. A *fusB* ARG, encoding fusidic acid resistance, was detected in a single isolate, but this agent was not tested in MIC analysis. Furthermore, a *vga(A)V* ARG, encoding tiamulin resistance, was detected in a single isolate. Tiamulin susceptibility was tested in the MIC analysis, but no ECOFF was available to infer resistance (Table 2). However, the MIC distribution for tiamulin demonstrated a bimodular distribution with a single isolate comprising a (potential) non-wildtype population, and this was the isolate where *vga(A)V* ARG was detected in its genome.

**Figure 1:**
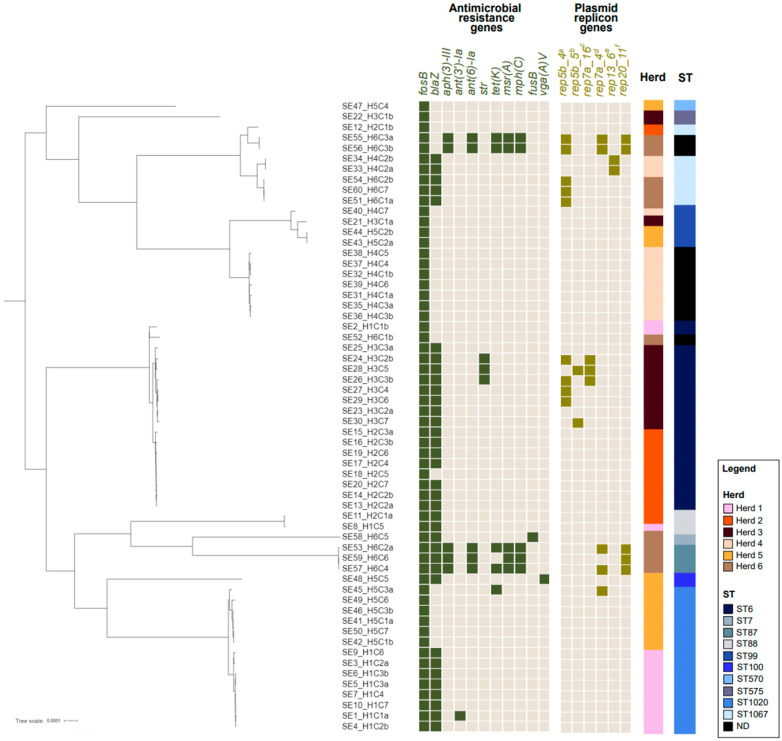
Core-genome phylogeny of 60 *S. epidermidis* isolates from SCM in Danish dairy cows. The isolates are named according to isolate number (SE1 to SE60), herd number (H1 to H6), and cow number (C1 to C7). The isolates are further named “a” or “b” in cases of cows with *S. epidermidis* in two quarters of the udder. The phylogenic tree is annotated with ARGs (dark green for positive) and plasmid replicon genes (light green for positive). ^a^ rep5b_4_SAP106B007(SAP106B). ^b^ rep5b_5_SAP108B006(SAP108B). ^c^ rep7a_4_repD(pK214), ^d^ rep7a_16_repC(Cassette), ^e^ rep13_6_rep(pLNU1), ^f^ rep20_11_repA(VRSAp). ST is presented in blue color scheme (ND = ST not determined).

The highest number of ARGs detected pr. genome was seven. Seven ARGs were detected in two genomes, which were both positive for *fosB* (fosfomycin), *blaZ* (penicillin), *aph(3′)-III* (amikacin), *ant6()-Ia* (streptomycin), *tet(K)* (tetracycline), *msaA*, and *mphC* (erythromycin). These two genomes were from 2/3 isolates deemed MDR according to the MIC analysis and originated from herd 6. Five out of ten isolates from herd 6 carried ARGs in their genomes, encoding resistance to more than three different antimicrobial classes, and isolates from herd 6 generally carried a higher number of ARGs in their genomes compared to isolates from the other study herds (Figure 1). Although a *blaZ* positive genome/isolate was found for all six herds, thus implying that penicillin resistant *S. epidermidis* strains are somewhat widely spread (Figure 1), most of the isolates from herd 4 and herd 5 were *blaZ* negative in their genomes, altogether indicating that the occurrence of resistance in *S. epidermidis* is rather herd specific.

### 2.3. Plasmid Replicons

The presence of plasmid replicon genes was investigated in the 60 *S. epidermidis* genomes to associate these findings with the observed AMR patterns.

Six different plasmid replicon genes were identified in total: rep5b_4_SAP106B007(SAP106B) was detected in 9/60 genomes (15.0%), rep5b_5_SAP108B006(SAP108B) was detected in 2/60 genomes (3.3%), rep7a_4_repD(pK214) was detected in 3/60 genomes (5.0%), rep7a_16_repC(Cassette) was detected in 5/60 genomes (8.3%)*,* rep13_6_rep(pLNU1) was detected in 2/60 genomes (3.3%), and, finally, rep20_11_repA(VRSAp) was detected in 5/60 genomes (8.3%).

A rep5b_4_SAP106B007(SAP106B) gene was detected in genomes of isolates from herd 6 (5/10 isolates) and herd 3 (4/10 isolates). A rep5b_5_SAP108B006(SAP108B) was detected in genomes of isolates from herd 2 (1/10 isolates) and herd 3 (1/10 isolates). A rep7a_4_repD(pK214) gene was detected in genomes of isolates solely from herd 3 (3/10 isolates). A rep7a_16_repC(Cassette) gene was detected in genomes of isolates from herd 5 (1/10 isolates) and herd 6 (4/10 isolates). A rep13_6_rep(pLNU1) gene was detected in two genomes of isolates, both from herd 4 (2/10). Finally, a rep20_11_repA(VRSAp) gene was detected in genomes of isolates from herd 6 exclusively (5/10 isolates).

All isolates with ≥1 plasmid replicons detected in their genomes further showed a resistant genotype with at least two different ARGs detected. The three isolates positive for a *str* ARG (encoding streptomycin resistance) were also positive for rep7a_4_repD(pK214) (Table 3) (Figure 1), and the genes were detected in the same contig. The five isolates positive for a *tetK* ARG (encoding tetracycline resistance) were all positive for rep7a_16_repC(Cassette) (Table 3) (Figure 1), and the genes were detected in the same contig. This indicates plasmid-mediated horizontal gene transfer (HGT) for streptomycin and tetracycline ARGs. For the remaining isolates having both ARGs and plasmid replicon genes detected in their genomes, the plasmid replicon genes were not located in the same contigs as the ARGs.

### 2.4. Strain Diversity

Based on their genomes, the strain diversity of the 60 *S. epidermidis* isolates was further explored. This involved identification of their sequence type (ST) and creating a phylogeny based on the core-genome (the core-genome represents the gene set shared by all the 60 isolates). The core-genome consisted of 1907 genes corresponding to 48.5% of the pangenome (the pan-genome represents the entire gene set of all 60 isolates). Thereby, around half of the genetic content was conserved among the 60 *S. epidermidis* isolates, which is slightly less when comparing to similar analyses of other NAS species from mastitis in dairy cows (e.g., ~70% for *S. rostri* [18], ~70% for *S. chromogenes* [19], and ~64% for *S. simulans* [19]).

Ten different STs were identified in total (Figure 1). The most prevalent ST was ST6 found for 17/60 isolates (28.3%), followed by ST1020 found for 14/60 isolates (23.3%). For 10/60 isolates (16.7%), an ST could not be identified.

The core-genome phylogeny demonstrated three major clades, each covering subclades with isolates clustering together according to their ST. Hence, three large subclades were seen in the phylogenetic tree, each representing isolates of, respectively, ST6, ST1020, and a clade with isolates with no predicted ST. The ST6 clade mainly covered isolates from herd 2 and herd 3, while the ST1020 clade included isolates from herd 1 and herd 5. The two herds represented within each of these clades were not located in same geographical region of Denmark (Table 1). Furthermore, the 60 *S. epidermidis* isolates originated from 42 cows, whereof 18 cows had *S. epidermidis* infection in two quarters (Table 1), and results showed that it was often the same *S. epidermidis* strain found in both quarters, since the two isolates (for each of such cases) mainly shared i. ST (10/18 cows), ii. clade in phylogeny (13/18 cows), and iii. resistance profile (11/18 cows) (Figure 1).

## 3. Discussion

Studies investigating prevalences of AMR among mastitis pathogens from Danish dairy cows are limited. Studies reporting both phenotypic and genotypic resistance prevalences (and their correlation) are even rarer. This study provides comprehensive insight into AMR patterns in 60 *S. epidermidis* isolates from cases of SCM in Danish dairy cows, with correspondence between phenotypic and genotypic resistance evaluated. This evaluation was based on MIC analysis and distribution of ARG from whole-genome sequencing and is the first Danish study of its kind. Increased knowledge within this field is needed as (1) *S. epidermidis* is one of the most common NAS associated with mastitis in dairy cows commonly treated with antimicrobials [5,6,7,8,15,20], (2) *S. epidermidis* has zoonotic potential and may transmit between hosts [21], and (3) *S. epidermidis* could act as a reservoir for ARGs and spread resistance to other pathogens [8]. Therefore, findings from this study contribute not only to veterinary practice in Denmark but also offer insights relevant to the broader One Health perspective.

According to MIC analysis and detection of phenotypic resistance, the highest resistance level was found towards penicillin (Table 2). Penicillin resistance was seen in 50.0% of the 60 *S. epidermidis* isolates analyzed (Table 2). Furthermore, at least one penicillin resistant isolate was found from each of the six herds (Figure 1). The high prevalence of penicillin resistance might reflect the predominant use of beta-lactams (including penicillin) for treatment of bovine mastitis in Denmark [5,7]. The present study therefore highlights the necessity of resistance testing before antimicrobial treatment in *S. epidermidis* mastitis and *S. epidermidis*-related dry-cow treatment. This can, however, be challenging if mastitis diagnostics are performed in laboratories/clinics using biochemical testing and bacterial culture methods only, as these diagnostics do not distinguish between NAS to the species level. Species-specific diagnostics are crucial, as resistance determination using MIC analysis involves the use of resistance breakpoints, which vary depending on the bacterial species, including different ECOFFs for different NAS [16]. Without accurate species identification, we risk using misleading resistance breakpoints, which can lead to incorrect interpretation of resistance. Incorrect interpretation of resistance might then lead to imprudent use of antimicrobials, which might again lead to an unsuccessful treatment in the particular cow but also to the further propagation of the resistance in the herd due to incognizant selection pressure on existing resistance-cases.

Regarding AMR prevalences, species-specific differences within NAS have been recorded [8,22,23], yet much of the existing literature reports resistance levels for NAS as a group rather than at the species level [9,12,13,14]. Interestingly, when reported at the species level, *S. epidermidis* appears to have higher resistance levels compared to other NAS species [8,22,24]. Aligning with our findings, high levels of penicillin resistance in *S. epidermidis* isolates from mastitis in dairy cows have been reported in studies from Sweden (~40.0%), Portugal (77.4%), and Korea (24.1%), among others [22,23,24]. In Denmark, similar trends were previously observed in studies by Aarestrup et al. and Chehabi et al., finding penicillin resistance levels of 36.1% and 22.4%, respectively [12,20]. However, it should be noted that Chehabi et al. [12] provided resistance data for a mix of NAS species (including *S. epidermidis*), while the study by Aarestrup et al. [20] is more than two decades old. Moreover, comparing resistance levels across studies requires caution, as methodologies and use of resistance breakpoints may differ between studies hampering true comparison. This issue is further addressed in the following sections.

According to the distribution of ARGs and detection of genotypic resistance, *fosB* was the most prevalent ARG found in all genomes of the 60 *S. epidermidis* isolates (Table 3) (Figure 1). Fosfomycin resistance encoded by the *fosB* gene has been reported among Gram-positive bacteria from various animals previously, however dairy cows remain less studied [25]. Although the use of fosfomycin in veterinary medicine is advised againsst in EU-countries [26], it remains an important antimicrobial in human medicine, reserved for infections caused by MDR bacteria [27]. Furthermore, the World Health Organization (WHO) classifies fosfomycin as one of the highest priority critically important antimicrobials (HPCIA) [28]. Therefore, a high prevalence of fosfomycin resistance in *S. epidermidis* is deeply concerning due to its possible spread to other pathogens or even hosts. However, in our study, we did not test for fosfomycin resistance phenotypically, as fosfomycin was not included in the applied MIC panel (Table 2). Hence, we do not know if or how the detected *fosB* gene is phenotypically expressed. Widerström et al. recently investigated phenotypic and genotypic fosfomycin resistance in *S. epidermidis* isolates from human prosthetic joint infections [29]. Similarly to our findings, when using the ResFinder database, they detected a *fosB* gene in all *S. epidermidis* genomes analyzed in their study (89/89 genomes) but only found a single isolate being fosfomycin resistant phenotypically (1/89 isolates) [29]. To explore genotypic fosfomycin resistance further, Widerström et al. applied two *fosB* reference sequences previously identified among *S. epidermidis* specifically and could only confirm the resistant genotype for the single isolate showing a resistant phenotype [29]. When using one of the same *fosB* reference sequences (NCBI Accession No. NG_050410.1) as Widerström et al. [29] for our 60 *S. epidermidis* genomes, we also could not verify this resistance. Therefore, like Widerström et al., we suggest being critical when evaluating findings of genotypic *fosB* resistance based on WGS data alone. Without corresponding phenotypic data, it remains uncertain whether the presence of *fosB* results in fosfomycin resistance in *S. epidermidis* [29]. These findings underscore the need for further research into the role of *fosB* and its potential implications for resistance in *S. epidermidis*.

Relying on Cohen’s kappa statistics, our data allowed us to study the correlation between phenotypic and genotypic resistance for penicillin, erythromycin, and tetracycline (Table 4). Cohen’s kappa analysis revealed an almost perfect agreement for penicillin and erythromycin resistance (Table 4). Regarding penicillin resistance, a *blaZ* ARG was detected in the genomes of five isolates, which did not show a penicillin resistant phenotype. Similar observations have been reported by others, and two possible explanations are suggested as underlying reasons of this phenomenon: (a) the ARG (*blaZ)* could be inactive/non-expressed, and/or (b) a misleading resistance breakpoint was used to interpret resistance phenotypically [14,15]. The latter (option b), however, seems unlikely in our study as five isolates were *blaZ*-positive yet penicillin susceptible and phenotypically all shared an identical MIC value (MIC 0.06 µg/mL) with the 30 isolates that were *blaZ*-negative and accordingly showed a penicillin susceptible phenotype. For tetracycline, Cohen’s kappa statistics revealed a substantial agreement between phenotypic and genotypic resistance, although three isolates were measured resistant in MIC analysis without having any ARGs conferring resistance detected in their genomes. This indicates the presence of non-identified resistance mechanism(s), but it was not explored further in our study.

Based on the MIC analysis, 3/60 isolates (5.0%) were deemed MDR, while 11/60 isolates (18.3%) had ARGs encoding resistance to ≥3 different antimicrobial classes detected in their genomes. This discrepancy probably reflects that we did not measure the occurrence of phenotypic resistance for 7/14 antimicrobials tested in MIC analysis (Table 2) due to the lack of ECOFFs for these seven agents. The general lack of resistance-breakpoints specific to cows and cow-related antimicrobial treatments remains a significant issue. That is both for resistance detection for surveillance purposes and for treatment guidance in diagnostic settings. Consequently, studies reporting resistance prevalences among mastitis pathogens are commonly using clinical breakpoints from diseases in other animals or humans or of other bacterial species [9,12,13,14]. This missing consensus in breakpoint usage can lead to the misinterpretation of resistance data and resistance levels when comparing results from studies employing different breakpoints. Therefore, the question arises whether it is advantageous to perform resistance surveillance based on the genotype rather than phenotype. In our study, this question is addressed by investigating phenotypic and genotypic resistance correlations. However, due to the lack of ECOFFs for many of the antimicrobials tested phenotypically, we were able to apply Cohen’s kappa statistics only to a limited number of antimicrobials (Table 4), where results did show almost perfect- or substantial agreement between phenotypic and genotypic resistance. Additionally, there was a 100% agreement between phenotypic and genotypic sensitivity to cefoxitin, ciprofloxacin, gentamicin, and trimethoprim. Furthermore, with the WGS data, ARGs were detected for antimicrobials to which resistance could not be measured phenotypically and further provided detailed insight into the occurrence of MDR (both due to lack of ECOFFs). Another advantage of WGS data are the ability to study how resistance might spread.

With a few exceptions, all *S. epidermidis* isolates having >1 ARG detected in their genomes also harbored minimum one plasmid replicon gene, thus pointing to plasmid-mediated HGT of ARGs among *S. epidermidis* (Figure 1). The isolates with most ARGs detected in their genomes (up to seven different ARGs) all came from herd 6 (Figure 1). Interestingly, the ARG-enriched isolates from herd 6 were displayed in two distinct clades in the core-genome phylogeny yet sharing identical resistance profile, and they were further all positive for rep20_11_repA(VRSAp) (Figure 1). While the rep20_11_repA(VRSAp) gene was not located in the same contig as any of the detected ARGs, we still suggest plasmid-mediated HGT, suggesting technical artifacts in genome assembly from short-read sequencing eventually having led to placement of, respectively, the ARGs and rep20_11_repA(VRSAp) in different contigs [30].

The WGS data further gave insight into the strain diversity among the 60 *S. epidermidis* isolates. Knowledge of strain diversity of the mastitis bacteria within a herd (e.g., *S. epidermidis*) can be informative regarding the study of transmission dynamics. Typically, low strain diversity suggests spread through contagious transmission (bacterial spread from infected to uninfected quarters) or through a shared environmental source [31]. On the other hand, high strain diversity is typically indicative of infections arising from independent, unrelated events [31]. The strain diversity among the 60 *S. epidermidis* isolates revealed that one primary strain predominated within each of the herds (Figure 1). Furthermore, for the cases of cows with two *S. epidermidis* infected quarters (n = 18 cows), it was most often the same *S. epidermidis* strain found in both quarters (same ST in 10/18 cases, same clade in phylogeny in 13/18 cases, and same resistance profile in 11/18 cases) (Figure 1). Altogether, this indicates that each herd has/had one major source of *S. epidermidis*. This could be an environmental source continually introducing *S. epidermidis* to the udder. For example, Thorberg et al. suggested human-to-cow transmission by physical contact between milker’s hands and cow udders during milking [21]. However, the observed patterns could also reflect dissemination of *S. epidermidis* within each herd through cow-to-cow transmission, as indicated for *S. epidermidis* in bovine mastitis by previous studies [32,33].

In this study, specific STs were not associated with specific resistance profiles. Instead, the occurrence of AMR seemed to vary from herd to herd. For example, the majority of isolates from herd 1 and herd 5 were of ST1020, but isolates from herd 1 were penicillin resistant while isolates from herd 5 were penicillin susceptible (Figure 1). This could reflect variation in treatment protocols within herds, but this was not investigated further as it was out of the frame for the current study. However, the considerable variation in AMR prevalences observed across herds highlights the importance of future studies involving a larger dataset from Danish dairy herds, as well as comparisons with international data. Of note, no MRSE was detected.

In conclusion, this study confirms occurrence of AMR in *S. epidermidis* from SCM in Danish dairy cows, with a high prevalence of penicillin resistance specifically. These findings support the necessity of testing for resistance prior to treatment to improve treatment outcome and ensure prudent use of antimicrobials. In particular, the high prevalence of penicillin resistance in *S. epidermidis* warns awareness towards dry-cow treatments. Species-specific diagnostics are essential for accurate resistance analysis and targeted control strategies. Furthermore, a *fosB* ARG was detected in all isolates (100.0%) and while this study did not test for fosfomycin resistance phenotypically, it calls for further investigation into the gene’s role in AMR among *S. epidermidis* and its potential implications in the One Health perspective. Finally, we emphasize the need for expanding AMR surveillance among other common mastitis pathogens.

## 4. Materials and Methods

### 4.1. Isolate Collection and Identification

The *S. epidermidis* isolates were previously collected as part of a large national study running from 2019 to 2021 in Denmark, which focused on exploring the distribution of bacteria associated with bovine SCM in Danish dairy cows (data to be published). In that study, quarter milk samples were collected from cows with SCM (somatic cell count (SCC) ≥200.000 cells/mL and no visible signs of IMI). For each milk sample, 10 µL was streaked onto a blood agar plate with 5% calf blood (SSI Diagnostica A/S, Hillerød, Denmark) and incubated at 37 °C for 24 and 48 h. Bacterial colonies were subcultured and then identified by matrix-assisted laser desorption ionization time of flight mass spectrometry (MALDI-TOF MS) to the bacterial species level (ID score ≥ 2.00) [34]. The identified isolates were frozen at −80 °C in LB broth with 15% glycerol. Milk samples with >2 bacterial species were omitted from the study as contaminated [3].

The number of isolates included in the present study was limited due to funding. The test herds were selected on the basis of their geographical distribution to ensure the widest spread while still representing the area in Denmark where the majority of milk production takes place (in Jutland, Denmark). The number of *S. epidermidis* isolates available for each herd was another selection criteria; for a herd to be included in the present study, it was required that *S. epidermidis* had been identified in two quarters simultaneously for a minimum of three cows. This was to allow studying resistance patterns and strain diversity of *S. epidermidis* on both a herd level and a cow level.

### 4.2. Antimicrobial Susceptibility Testing

The MIC for each isolate towards 14 antimicrobial agents (Table 2) was determined by the broth microdilution method using a semiautomatic system (SensiTitre, Trek Diagnostic Systems Ltd., UK) in accordance with guidelines from the Clinical Laboratory Standards Institute (CLSI) [35]. The MIC panel applied was custom made and has previously been used for resistance surveillance of veterinary Gram-positive bacteria, including staphylococci from bovine mastitis [12]. The 14 antimicrobials and their test ranges are presented in Table 2. Interpretation of the observed MIC values was carried out using ECOFFs when available (Table 2) [16]. The MIC50 and MIC90 were assessed representing the MIC value of which, respectively, 50% and 90% of the isolates had their growth inhibited by the antimicrobial agent [36].

### 4.3. Whole-Genome Sequencing and Bioinformatic Tools

The isolates were submitted to Novogene (Novogene (UK), Co., Ltd., Cambridge, UK) for DNA extraction and whole-genome sequencing using an Illumina NovoSeq6000 platform generating 2 × 150 bp paired-end reads. The raw reads were inspected for quality using FastQC (v.0.11.9) and thereafter assembled applying SPAdes (v.3.13.1) [37]. QUAST (v.5.0.2) was used to evaluate the quality of the assembled genomes prior to the downstream analyses [38]. These involved detection of (i) ARGs using Abricate (v.1.0.1) and ResFinder [39,40] (https://github.com/tseemann/abricate, accessed on 9 October 2023). Presence of *fosB* reference sequence, NCBI Accession No. NG_050410.1, was investigated using NCBI’s BLASTN online service (https://blast.ncbi.nlm.nih.gov/Blast.cgi?PROGRAM=blastn&BLAST_SPEC=GeoBlast&PAGE_TYPE=BlastSearch, accessed on 18 November 2024), (ii) plasmid replicon genes using Abricate (v.1.0.1) and PlasmidFinder [40,41], and (iii) ST using: https://github.com/tseemann/mlst, accessed on 12 October 2023. Annotation was performed using prokka (v.1.14.6) [42]. The core-genome was determined using roary (v.3.13.0) [43] using the GFF3 files obtained from the prokka annotation. RAxML-NG (v.0.9.0) [44] was used to construct a phylogenetic tree based on the core-genome alignment obtained from roary. Finally, a graphic representation of the core-genome phylogeny was conducted in iTol [45] and Inkscape (https://inkscape.org/).

### 4.4. Stastical Analysis

The correlation between phenotypic and genotypic resistance was evaluated using Cohen’s kappa statistics in RStudio (v.4.2.3) using the vcd package (v.1.4-13). The value of kappa (κ) was interpreted according to level of agreement, using these ranges: κ ≤ 0 indicating no agreement, κ = 0.01–0.20 indicating no to slight agreement, κ = 0.21–0.40 indicating fair agreement, κ = 0.41–0.60 indicating moderate agreement, κ = 0.61–0.80 indicating substantial agreement, and κ = 0.81–1.00 indicating an almost perfect agreement [46].

## Figures and Tables

**Table 1:** Overview of the 60 *S. epidermidis* isolates analyzed in the current study. The isolates originate from Danish dairy cows with SCM identified during 2019–2020.

Herd	Geographical Region of Denmark	n Isolates	n Cows	n Cows with One Infected Quarter of the Udder	n Cows with Two Infected Quarters of the Udder
Herd 1	Central	10	7	4	3
Herd 2	Central	10	7	4	3
Herd 3	South	10	7	4	3
Herd 4	North	10	7	4	3
Herd 5	North	10	7	4	3
Herd 6	Central	10	7	4	3
Sum		60	42	24	18

**Table 2:** Distribution of minimum inhibitory concentrations (MICs) of 60 *S. epidermidis* isolates from SCM in Danish dairy cows towards 14 antimicrobials.

	Minimum Inhibitory Concentration (MIC) (µg/mL)																	
Agent	0.06	0.12	0.25	0.5	1	2	4	8	16	32	64	128	256	512		MIC50	MIC90	%R
Cefoxitin (FOX)					7	51	2									2	2	0.0%
Chloramphenicol (CHL)						2	47	11								4	8	ND
Ciprofloxacin (CIP)		11	48	1												0.25	0.25	0.0%
Erythromycin (ERY)			48	6	1				5							0.25	0.5	8.3%
Florfenicol (FFN)					2	43	14	1								2	4	ND
Gentamicin (GEN)			60													0.25	0.25	0.0%
Penicillin (PEN)	30	6	13	10	1											0.06	0.5	50.0%
Spectinomycin (SPE)									2	20	38					64	64	ND
Streptomycin (STR)							41	10	3	1	1	4				4	16	ND
Sulfamethoxazole (SMX)										55			2	3		32	32	ND
Tetracycline (TET)				50	2	3				2	3					0.5	2	13.3%
Tiamulin (TIA)			7	51	1				1							0.5	0.5	ND
Trimethoprim (TMP)				58	2											0.5	0.5	0.0%
TMP+SMX			60													0.25	0.25	ND

For each of the 14 antimicrobials tested (left), the white fields to the right indicate the dilution ranges tested (µg/mL) (e.g., cefoxitin was tested in concentrations ranging from 0.5 µg/mL to 32 µg/mL). Values in each field represent the number of isolates with their respective MIC value (e.g., for cefoxitin, 7 isolates had a MIC value of 1 µg/mL). For each antimicrobial and test range, numbers given in the lowest test concentration represent the number of isolates with a MIC value ≤ the lowest concentration tested (e.g., for ciprofloxacin, 11 isolates had a MIC value ≤ 0.12 µg/mL). Numbers in the first field above the highest concentration in the test range (numbers outside the white fields) represent the number of isolates with a MIC value > the highest concentration tested (e.g., for erythromycin, 5 isolates had a MIC value > 8 µg/mL). The vertical line within the test range represents the ECOFF applied to interpret phenotypic resistance (%R) (e.g., an ECOFF of 4 µg/mL was used for cefoxitin).

**Table 3:** Detection of antimicrobial resistance genes (ARGs) among 60 *S. epidermidis* isolates from SCM in Danish dairy cows in 2019–2020.

Class	Agents ^1^	Gene	n Isolates (%)
Phosphonic acid	Fosfomycin	*fosB*	60 (100.0%)
Beta-lactams	Amoxicillin, ampicillin, penicillin, and piperacillin	*blaZ*	35 (58.3%)
Aminoglycosides	Amikacin,	*aph(3′)-III*	5 (8.3%)
	streptomycin	*ant(6)-Ia*	5 (8.3%)
		*str*	3 (5.0%)
		*ant(3″)-Ia*	1 (1.7%)
Macrolides	Erythromycin, azithromycin, telithromycin, quinupristin, pristinamycin, and virginiamycin	*msr(A)*	5 (8.3%)
	Erythromycin, spiramycin, telithromycin	*mph(C)*	5 (8.3%)
Tetracyclines	doxycycline, and tetracycline	*tet(K)*	5 (8.3%)
Lincosamides	Lincomycin, dalfopristin, pristinamycin, virginiamycin, and tiamulin	*vga(A)V*	1 (1.7%)
Fusidane	Fusidic acid	*fusB*	1 (1.7%)

^1^ Resistant phenotype according to ResFinder.

**Table 4:** Cohen’s kappa statistics evaluating the agreement between phenotypic and genotypic resistance in 60 *S. epidermidis* isolates from SCM in Danish dairy cows from 2019–2020.

Class	Agents Tested in MIC	No. of Phenotypically Resistant Isolates	No. of Genomes Positive for ≥1 Corresponding ARG	Kappa	*p*-Value
Beta-lactams	Penicillin	30	35	0.83	<0.01
Macrolides	Erythromycin	5	5	1	0.00
Tetracyclines	Tetracycline	8	5	0.74	<0.01

CHL, FFN, SPE, STR, SMX, TIA, and TMP+SMX are not reported since no ECOFFs are available to measure phenotypic resistance for these antimicrobials (Table 2). FOX, CIP, GEN, and TMP results are not reported since none of the 60 *S. epidermidis* isolates were phenotypically resistant to these antimicrobials, nor were any ARGs conferring resistance to these agents detected (Table S3). Fosfomycin, amikacin, and fusidic acid are not reported since they were not tested in MIC analysis, although ARGs conferring resistance to these agents were detected (Table 3).

## Data Availability

The original contributions presented in the study are included in the article/Supplementary Material, further inquiries can be directed to the corresponding author.

## Supplementary Materials

The following supporting information can be downloaded at: https://www.mdpi.com/article/10.3390/antibiotics14010067/s1, Table S1: Bacteria identified in mixed-growth samples; Table S2: *S. epidermidis* isolates from bovine subclinical mastitis in Danish dairy cows; Table S3: Additional information regarding Cohen’s kappa statistics for evaluating the agreement between phenotypic and genotypic resistance.

## References

[1] World Health Organization (WHO) Antimicrobial Resistance. https://www.who.int/news-room/fact-sheets/detail/antimicrobial-resistance.

[2] Rasmussen P., Barkema H.W., Osei P.P., Taylor J., Shaw A.P., Conrady B., Chaters G., Muñoz V., Hall D.C., Apenteng O.O. (2024). Global losses due to dairy cattle diseases: A comorbidity-adjusted economic analysis. J. Dairy Sci..

[3] Bradley A.J. (2002). Bovine Mastitis: An Evolving Disease. Vet. J..

[4] Bulletin of the International Dairy Federation 448/2011 Suggested Interpretation of Mastitis Terminology (Revision of Bulletin of IDF N◦ 338/1999). List of Terms and Interpretations. https://shop.fil-idf.org/products/suggested-interpretation-of-mastitis-terminology-revision-of-bulletin-of-idf-n-3381999.

[5] DANMAP 2023 Use of Antimicrobial Agents and Occurrence of Antimicrobial Resistance in Bacteria from Food Animals, Food and Humans in Denmark. 4.3.2 Antimicrobial Consumption in Cattle. https://www.danmap.org/reports/2023/kap-4.

[6] Pyörälä S. (2009). Treatment of mastitis during lactation. Ir. Vet. J..

[7] Wilm J., Svennesen L., Eriksen E.Ø., Halasa T., Krömker V. (2021). Veterinary Treatment Approach and Antibiotic Usage for Clinical Mastitis in Danish Dairy Herds. Antibiotics.

[8] De Buck J., Ha V., Naushad S., Nobrega D.B., Luby C., Middleton J.R., De Vliegher S., Barkema H.W. (2021). Non-aureus Staphylococci and Bovine Udder Health: Current Understanding and Knowledge Gaps. Front. Vet. Sci..

[9] Pitkälä A., Haveri M., Pyörälä S., Myllys V., Honkanen-Buzalski T. (2004). Bovine mastitis in Finland 2001–Prevalence, distribution of bacteria, and antimicrobial resistance. J. Dairy Sci..

[10] Astrup L.B. (2024). Personal communication.

[11] Freu G., Gioia G., Gross B., Biscarini F., Virkler P., Watters R., Addis M.F., Franklin-Guild R.J., Runyan J., Masroure A.J. (2024). Frequency of non-aureus staphylococci and mammaliicocci species isolated from quarter clinical mastitis: A 6-year retrospective study. J. Dairy Sci..

[12] Chehabi C.N., Nonnemann B., Astrup L.B., Farre M., Pedersen K. (2019). In vitro Antimicrobial Resistance of Causative Agents to Clinical Mastitis in Danish Dairy Cows. Foodborne Pathog. Dis..

[13] de Jong A., Garch F.E., Simjee S., Moyaert H., Rose M., Youala M., Siegwart E. (2018). Monitoring of antimicrobial susceptibility of udder pathogens recovered from cases of clinical mastitis in dairy cows across Europe: VetPath results. Vet. Microbiol..

[14] Ruegg P.L., Oliveira L., Jin W., Okwumabua O. (2015). Phenotypic antimicrobial susceptibility and occurrence of selected resistance genes in gram-positive mastitis pathogens isolated from Wisconsin dairy cows. J. Dairy Sci..

[15] Bolte J., Zhang Y., Wente N., Mahmmod Y.S., Svennesen L., Krömker V. (2020). Comparison of phenotypic and genotypic antimicrobial resistance patterns associated with Staphylococcus aureus mastitis in German and Danish dairy cows. J. Dairy Sci..

[16] European Committee on Antimicrobial Susceptibility Testing EUCAST: Epidemiological Cut-Off Values (ECOFFs). https://www.EUCAST.com.

[17] Magiorakos A.-P., Srinivasan A., Carey R.B., Carmeli Y., Falagas M.E., Giske C.G., Harbarth S., Hindler J.F., Kahlmeter G., Olsson-Lilj B. (2012). Multidrug-resistant, extensively drug-resistant and pandrug-resistant bacteria: An international expert proposal for interim standard definitions for acquired resistance. Clin. Microbiol. Infect..

[18] Kløve D.C., Farre M., Strube M.L., Astrup L.B. (2023). Comparative Genomics of Staphylococcus rostri, an Undescribed Bacterium Isolated from Dairy Mastitis. Vet. Sci..

[19] Åvall-Jääskeläinen S., Taponen S., Kant R., Paulin L., Blom J., Palva A., Koort J. (2018). Comparative genome analysis of 24 bovine-associated Staphylococcus isolates with special focus on the putative virulence genes. PeerJ.

[20] Aarestrup F.M., Wegener H.C., Rosdahl V.T., Jensen N.E. (1995). Staphylococcal and other Bacterial Species Associated with Intramammary Infections in Danish Dairy Herds. Acta Vet. Scand..

[21] Thorberg B.M., Kuhn I., Aarestrup F.M., Brandstrom B., Jonsson P., Danielsson-Tharn M.L. (2006). Pheno–And genotyping of Staphylococcus epidermidis isolated from bovine milk and human skin. Vet. Microbiol..

[22] Persson Waller K., Aspán A., Nyman A., Persson Y., Grönlund Andersson U. (2011). CNS species and antimicrobial resistance in clinical and subclinical bovine mastitis. Vet. Microbiol..

[23] Kim S.J., Moon D.C., Park S.C., Kang H.Y., Na S.H., Lim S.K. (2019). Antimicrobial resistance and genetic characterization of coagulase-negative staphylococci from bovine mastitis milk samples in Korea. J. Dairy Sci..

[24] Nunes S.F., Bexiga R., Cavaco L.M., Vilela C.L. (2007). Technical Note: Antimicrobial Susceptibility of Portuguese Isolates of Staphylococcus aureus and Staphylococcus epidermidis in Subclinical Bovine Mastitis. J. Dairy Sci..

[25] Lysitsas M., Chatzipanagiotidou I., Billinis C., Valiakos G. (2023). Fosfomycin Resistance in Bacteria Isolated from Companion Animals (Dogs and Cats). Vet. Sci..

[26] European Medicines Agency (EMA) (2019). Categorisation of Antibiotics in the European Union. https://www.ema.europa.eu/en/documents/report/categorisation-antibiotics-european-union-answer-request-european-commission-updating-scientific-advice-impact-public-health-and-animal-health-use-antibiotics-animals_en.pdf.

[27] World Health Organization (2023). WHO AWaRe Classification of Antibiotics for Evaluation and Monitoring of Use. https://www.who.int/publications/i/item/WHO-MHP-HPS-EML-2023.04.

[28] World Health Organization (2024). WHO’s List of Medically Important Antimicrobials: A Risk Management Tool for Mitigating Antimicrobial Resistance Due to Non-Human Use.

[29] Widerström R., Aarris M., Jacobsson S., Stegger M., Söderquist B., Månsson E. (2024). Probing fosfomycin’s potential: A study on susceptibility testing and resistance in *Staphylococcus epidermidis* from prosthetic joint infections. J. Antimicrob. Chemother..

[30] Petrin S., Orsini M., Massaro A., Olsen J.E., Barco L., Losasso C. (2023). Phenotypic and genotypic antimicrobial resistance correlation and plasmid characterization in *Salmonella* spp. isolates from Italy reveal high heterogeneity among serovars. Front. Public Heal..

[31] Woudstra S., Wente N., Zhang Y., Leimbach S., Gussmann M.K., Kirkeby C., Krömker V. (2023). Strain diversity and infection durations of *Staphylococcus* spp. and *Streptococcus* spp. causing intramammary infections in dairy cows. J. Dairy Sci..

[32] Sawant A.A., Gillespie B.E., Oliver S.P. (2009). Antimicrobial susceptibility of coagulase-negative *Staphylococcus* species isolated from bovine milk. Vet. Microbiol..

[33] Piessens V., De Vliegher S., Verbist B., Braem G., Van Nuffel A., De Vuyst L., Heyndrickx M., Van Coillie E. (2012). Intra-species diversity and epidemiology varies among coagulase-negative *Staphylococcus* species causing bovine intramammary infections. Vet. Microbiol..

[34] Bizzini A., Durussel C., Bille J., Greub G., Prod’hom G. (2010). Performance of matrix-assisted laser desorption ionization-time of flight mass spectrometry for identification of bacterial strains routinely isolated in a Clinical Microbiology Laboratory. J. Clin. Microbiol..

[35] Clinical and Laboratory Standards Institute (CLSI) (2018). Performance Standards for Antimicrobial Disk and Dilution Susceptibility Tests for Bacteria Isolated from Animals.

[36] Clinical and Laboratory Standards Institute (CLSI) (2024). Performance Standards for Antimicrobial Disk and Dilution Susceptibility Tests for Bacteria Isolated from Animals.

[37] Nurk S., Bankevich A., Antipov D., Gurevich A., Korobeynikov A., Lapidus A., Prjibelsky A., Pyshkin A., Sirotkin A., Sirotkin Y. (2013). Assembling Single-Cell Genomes and Mini-Metagenomes from Chimeric MDA Products. J. Comput. Biol..

[38] Gurevich A., Saveliev V., Vyahhi N., Tesler G. (2013). QUAST: Quality assessment tool for genome assemblies. Bioinformatics.

[39] Bortolaia V., Kaas R.F., Ruppe E., Roberts M.C., Schwarz S., Cattoir V., Philippon A., Allesoe R.L., Rebelo A.R., Florensa A.R. (2020). ResFinder 4.0 for predictions of phenotypes from genotypes. J. Antimicrob. Chemother..

[40] Camacho C., Coulouris G., Avagyan V., Ma N., Papadopoulos J., Bealer K., Madden T.L. (2009). BLAST+: Architecture and applications. BMC Bioinform..

[41] Carattoli A., Zankari E., Garcia-Fernandez A., Larsen M.V., Lund O., Villa L., Aarestrup F.M., Hasman H. (2014). PlasmidFinder and pMLST: In silico detection and typing of plasmids. Antimicrob. Agents Chemother..

[42] Seemann T. (2014). Prokka: Rapid prokaryotic genome annotation. Bioinformatics.

[43] Page A.J., Cummins C.A., Hunt M., Wong V.K., Reuter S., Holden M.T.G., Fookes M., Falush D., Keane J.A., Parkhill J. (2015). Roary: Rapid large-scale prokaryote pan genome analysis. Bioinformatics.

[44] Kozlov A.M., Darriba D., Flouri T., Morel B., Stamatakis A. (2019). RAxML-NG: A fast, scalable, and user-friendly tool for maximum likelihood phylogenetic inference. Bioinformatics.

[45] Letunic I., Bork P. (2021). Interactive Tree of Life (iTOL) v5: An online tool for phylogenetic tree display and annotation. Nucleic Acids Res..

[46] McHugh M.L. (2012). Interrater reliability: The kappa statistic. Biochem Med..

