# Supplementary material for: Mapping Antimicrobial Resistance in Staphylococcus epidermidis Isolates from Subclinical Mastitis in Danish Dairy Cows
Source: Antibiotics (Basel). 2025 Jan 10;14(1):67. doi: 10.3390/antibiotics14010067 (PMC11761952; doi:10.3390/antibiotics14010067)
Supplement: Supplementary file 1 [file antibiotics-14-00067-s001.zip › Table S1..docx]

**Table S1.** Bacteria identified in mixed-growth samples

| **Bacterial species**^1^ | **Prevalence (%)** |
| --- | --- |
| *Aerococcus viridans* | 3 (13.64) |
| *Corynebacteriumm amycolatum* | 1 (4.55) |
| *Corynebacterium frankeforstense* | 1 (4.55) |
| *Enterococcus faecalis* | 2 (9.09) |
| *Enterococcus faecium* | 2 (9.09) |
| *Micrococcus luteus* | 2 (9.09) |
| *Staphylococcus arlettae* | 1 (4.55) |
| *Staphylococcus chromogenes* | 1 (4.55) |
| *Staphylococcus haemolyticus* | 1 (4.55) |
| *Staphylococcus hominis* | 1 (4.55) |
| *Streptococcus hyovaginalis* | 1 (4.55) |
| *Staphylococcus rostri* | 5 (22.73) |
| *Streptococcus uberis* | 1 (4.55) |
| **SUM** | 22 (100.0%) |

^1^Bacterial species identified in combination with *S. epidermidis* isolates. 36.7% (n=22) of the *S. epidermidis* isolates were found in mixed culture.
