# Supplementary material for: Mapping Antimicrobial Resistance in Staphylococcus epidermidis Isolates from Subclinical Mastitis in Danish Dairy Cows
Source: Antibiotics (Basel). 2025 Jan 10;14(1):67. doi: 10.3390/antibiotics14010067 (PMC11761952; doi:10.3390/antibiotics14010067)
Supplement: Supplementary file 1 [file antibiotics-14-00067-s001.zip › Table S2..docx]

**Table S2.** *S. epidermidis* isolates from bovine subclinical mastitis in Danish dairy cows

| **Isolate^1^** | **Herd** | **Geographical**  **region of herd** | **Year of sample collection** | **Pure culture/**  **mixed culture** | **Assembly metrics:** |  |  |  | **Phenotypic**  **resistance**  **profile** | **Genotypic resistance profile** | **Plasmid replicon gene(s)** |
| --- | --- | --- | --- | --- | --- | --- | --- | --- | --- | --- | --- |
|  |  |  |  |  | Assembly  size (Mb) | n  contigs | GC content (%) | N50 (Kb) |  |  |  |
| SE1_H1C1a | Herd 1 | Central Region  of Denmark | 2019 | *Staphylococcus arlettae* | 2.41 | 49 | 32.12 | 183.72 | Penicillin  (MIC 0.5µg/mL) | *ant(3'')-Ia*  *fosB*  *blaZ* |  |
| SE2_H1C1b | Herd 1 | Central Region  of Denmark | 2019 | Pure | 2.39 | 31 | 31.96 | 183.72 |  | *fosB* |  |
| SE3_H1C2a | Herd 1 | Central Region  of Denmark | 2019 | *Aerococcus viridans* | 2.43 | 39 | 31.94 | 135.64 | Penicillin  (MIC 0.5µg/mL) | *fosB*  *blaZ* |  |
| SE4_H1C2b | Herd 1 | Central Region of Denmark | 2019 | Pure | 2.41 | 30 | 32.07 | 297.46 | Penicillin  (MIC 0.25µg/mL) | *fosB*  *blaZ* |  |
| SE5_H1C3a | Herd 1 | Central Region of Denmark | 2019 | *Staphylococcus chromogenes* | 2.43 | 29 | 32.04 | 203.71 | Penicillin  (MIC 0.5µg/mL) | *fosB*  *blaZ* |  |
| SE6_H1C3b | Herd 1 | Central Region of Denmark | 2019 | Pure | 2.36 | 25 | 32.01 | 203.71 | Penicillin  (MIC 0.5µg/mL) | *fosB*  *blaZ* |  |
| SE7_H1C4 | Herd 1 | Central Region of Denmark | 2019 | *Enterococcus faecalis* | 2.41 | 22 | 32.06 | 203.71 | Penicillin  (MIC 0.5µg/mL) | *fosB*  *blaZ* |  |
| SE8_H1C5 | Herd 1 | Central Region of Denmark | 2019 | Pure | 2.41 | 26 | 32.05 | 184.17 | Penicillin  (MIC 0.25µg/mL).  Tetracycline  (MIC 2µg/mL) | *blaZ*  *fosB* |  |
| SE9_H1C6 | Herd 1 | Central Region of Denmark | 2019 | *Enterococcus faecalis* | 2.41 | 26 | 32.05 | 203.70 | Penicillin  (MIC 0.25µg/mL) | *blaZ*  *fosB* |  |
| SE10_H1C7 | Herd 1 | Central Region of Denmark | 2019 | *Aerococcus viridans* | 2.41 | 26 | 32.07 | 203.71 | Penicillin  (MIC 0.5µg/mL) | *fosB*  *blaZ* |  |
| SE11_H2C1a | Herd 2 | Central Region of Denmark | 2020 | Pure | 2.41 | 26 | 32.05 | 203.71 | Penicillin  (MIC 0.25µg/mL).  Tetracycline  (MIC 2µg/mL) | *fosB*  *blaZ* |  |
| SE12_H2C1b | Herd 2 | Central Region of Denmark | 2020 | Pure | 2.37 | 29 | 32.03 | 203.79 |  | *fosB* |  |
| SE13_H2C2a | Herd 2 | Central Region of Denmark | 2020 | *Corynebacterium frankeforstense* | 2.41 | 23 | 32.04 | 204.03 | Penicillin  (MIC 0.25µg/mL) | *fosB*  *blaZ* |  |
| SE14_H2C2b | Herd 2 | Central Region of Denmark | 2020 | Pure | 2.42 | 27 | 32.07 | 226.49 | Penicillin  (MIC 0.25µg/mL) | *fosB*  *blaZ* |  |
| SE15_H2C3a | Herd 2 | Central Region of Denmark | 2020 | *Aerococcus viridans* | 2.44 | 28 | 32.06 | 211.14 | Penicillin  (MIC 0.25µg/mL) | *fosB*  *blaZ* |  |
| SE16_H2C3b | Herd 2 | Central Region of Denmark | 2020 | Pure | 2.45 | 26 | 32.02 | 203.67 | Penicillin  (MIC 0.25µg/mL) | *fosB*  *blaZ* |  |
| SE17_H2C4 | Herd 2 | Central Region of Denmark | 2020 | Pure | 2.41 | 26 | 32.04 | 184.17 | Penicillin  (MIC 0.25µg/mL) | *fosB*  *blaZ* |  |
| SE18_H2C5 | Herd 2 | Central Region of Denmark | 2020 | Pure | 2.39 | 28 | 31.98 | 184.17 |  | *fosB* |  |
| SE19_H2C6 | Herd 2 | Central Region of Denmark | 2020 | Pure | 2.45 | 29 | 32.02 | 184.17 | Penicillin  (MIC 0.5µg/mL) | *fosB*  *blaZ* |  |
| SE20_H2C7 | Herd 2 | Central Region of Denmark | 2020 | Pure | 2.40 | 27 | 31.99 | 204.13 | Penicillin  (MIC 0.12µg/mL) | *fosB*  *blaZ* |  |
| SE21_H3C1a | Herd 3 | Southern Region of Denmark | 2019 | Pure | 2.42 | 28 | 32.04 | 203.68 |  | \| *fosB* \| \| --- \| \|  \| |  |
| SE22_H3C1b | Herd 3 | Southern Region of Denmark | 2019 | Pure | 2.40 | 23 | 32.00 | 203.68 |  | *fosB* |  |
| SE23_H3C2a | Herd 3 | Southern Region of Denmark | 2019 | Pure | 2.38 | 29 | 31.97 | 183.72 |  | *fosB*  *blaZ* |  |
| SE24_H3C2b | Herd 3 | Southern Region of Denmark | 2019 | *Corynebacteriumm amycolatum* | 2.40 | 22 | 31.99 | 203.99 |  | *str*  *fosB*  *blaZ* | *rep5b*  *rep7a* |
| SE25_H3C3a | Herd 3 | Southern Region of Denmark | 2019 | Pure | 2.46 | 26 | 31.98 | 320.32 |  | *fosB*  *blaZ* |  |
| SE26_H3C3b | Herd 3 | Southern Region of Denmark | 2019 | *Enterococcus faecium* | 2.44 | 26 | 31.97 | 235.26 | Penicillin  (MIC 0.12µg/mL) | *str*  *fosB*  *blaZ* | *rep5b*  *rep7a* |
| SE27_H3C4 | Herd 3 | Southern Region of Denmark | 2019 | Pure | 2.48 | 43 | 31.87 | 128.16 | Penicillin  (MIC 0.12µg/mL) | *fosB*  *blaZ* | *rep5b* |
| SE28_H3C5 | Herd 3 | Southern Region of Denmark | 2019 | Pure | 2.48 | 41 | 31.87 | 139.57 | Penicillin  (MIC 0.12µg/mL) | *str*  *fosB*  *blaZ* | *rep5b*  *rep7a* |
| SE29_H3C6 | Herd 3 | Southern Region of Denmark | 2019 | Pure | 2.50 | 27 | 31.98 | 321.05 |  | *fosB*  *blaZ* | *rep5b* |
| SE30_H3C7 | Herd 3 | Southern Region of Denmark | 2019 | Pure | 2.49 | 26 | 31.99 | 320.32 | Penicillin  (MIC 0.12µg/mL) | *fosB*  *blaZ* | *rep5b* |
| SE31_H4C1a | Herd 4 | Northen Region of Denmark | 2020 | *Staphylococcus rostri* | 2.48 | 26 | 31.98 | 320.47 |  | *fosB* |  |
| SE32_H4C1b | Herd 4 | Northen Region of Denmark | 2020 | Pure | 2.48 | 29 | 31.98 | 320.32 |  | *fosB* |  |
| SE33_H4C2a | Herd 4 | Northen Region of Denmark | 2020 | Pure | 2.50 | 27 | 31.98 | 320.91 |  | *fosB*  *blaZ* | *rep13* |
| SE34_H4C2b | Herd 4 | Northen Region of Denmark | 2020 | *Staphylococcus rostri* | 2.39 | 32 | 31.97 | 204.03 | Penicillin  (MIC 0.12µg/mL) | *fosB*  *blaZ* | *rep13* |
| SE35_H4C3a | Herd 4 | Northen Region of Denmark | 2020 | Pure | 2.48 | 23 | 32.05 | 234.82 |  | *fosB* |  |
| SE36_H4C3b | Herd 4 | Northen Region of Denmark | 2020 | Pure | 2.38 | 36 | 31.99 | 203.97 |  | *fosB* |  |
| SE37_H4C4 | Herd 4 | Northen Region of Denmark | 2020 | *Staphylococcus rostri* | 2.37 | 26 | 31.99 | 183.73 |  | *fosB* |  |
| SE38_H4C5 | Herd 4 | Northen Region of Denmark | 2020 | *Staphylococcus rostri* | 2.48 | 26 | 32.05 | 234.91 |  | *fosB* |  |
| SE39_H4C6 | Herd 4 | Northen Region of Denmark | 2020 | *Staphylococcus rostri* | 2.49 | 27 | 32.07 | 234.91 |  | *fosB* |  |
| SE40_H4C7 | Herd 4 | Northen Region of Denmark | 2020 | *Streptococcus hyovaginalis* | 2.38 | 29 | 31.99 | 183.73 |  | *fosB* |  |
| SE41_H5C1a | Herd 5 | Northen Region of Denmark | 2020 | Pure | 2.37 | 27 | 31.99 | 183.89 |  | *fosB* |  |
| SE42_H5C1b | Herd 5 | Northen Region of Denmark | 2020 | Pure | 2.44 | 29 | 32.02 | 177.60 |  | *fosB* |  |
| SE43_H5C2a | Herd 5 | Northen Region of Denmark | 2020 | *Micrococcus luteus* | 2.42 | 36 | 32.08 | 160.38 |  | *fosB* |  |
| SE44_H5C2b | Herd 5 | Northen Region of Denmark | 2020 | *Micrococcus luteus* | 2.38 | 26 | 31.99 | 183.89 |  | *fosB* |  |
| SE45_H5C3a | Herd 5 | Northen Region of Denmark | 2020 | Pure | 2.38 | 29 | 31.97 | 204.03 | Tetracycline (MIC >32µg/mL) | *tet(K)*  *fosB* | *rep7a* |
| SE46_H5C3b | Herd 5 | Northen Region of Denmark | 2020 | Pure | 2.37 | 25 | 31.99 | 203.97 |  | *fosB* |  |
| SE47_H5C4 | Herd 5 | Northen Region of Denmark | 2020 | Pure | 2.51 | 40 | 31.93 | 270.69 |  | *fosB* |  |
| SE48_H5C5 | Herd 5 | Northen Region of Denmark | 2020 | Pure | 2.42 | 34 | 32.16 | 210.27 | Penicillin  (MIC 0.5µg/mL) | *fosB*  *vga(A)V*  *blaZ* |  |
| SE49_H5C6 | Herd 5 | Northen Region of Denmark | 2020 | *Staphylococcus hominis* | 2.46 | 43 | 31.93 | 173.52 |  | *fosB* |  |
| SE50_H5C7 | Herd 5 | Northen Region of Denmark | 2020 | Pure | 2.54 | 48 | 31.95 | 270.34 |  | *fosB* |  |
| SE51_H6C1a | Herd 6 | Central Region of Denmark | 2020 | Pure | 2.45 | 65 | 32.14 | 117.40 | Penicillin  (MIC 0.25µg/mL) | *fosB*  *blaZ* | *rep5b* |
| SE52_H6C1b | Herd 6 | Central Region of Denmark | 2020 | Pure | 2.44 | 60 | 32.14 | 121.86 |  | *fosB* |  |
| SE53_H6C2a | Herd 6 | Central Region of Denmark | 2020 | Pure | 2.46 | 38 | 31.93 | 201.02 | Penicillin  (MIC 0.5µg/mL).  erythromycin  (MIC >16µg/mL).  tetracycline  (MIC >32µg/mL) | *fosB*  *tet(K)*  *msr(A)*  *mph(C)*  *aph(3')-III*  *ant(6)-Ia*  *blaZ* | *rep20*  *rep7a* |
| SE54_H6C2b | Herd 6 | Central Region of Denmark | 2020 | Pure | 2.44 | 29 | 32.07 | 165.06 | Penicillin  (MIC 0.5µg/mL) | *fosB*  *blaZ* | *rep5b* |
| SE55_H6C3a | Herd 6 | Central Region of Denmark | 2020 | *Streptococcus uberis* | 2.41 | 36 | 31.98 | 201.02 | Erythromycin  (MIC >16µg/mL).  tetracycline  (MIC 32µg/mL) | *fosB*  *tet(K)*  *mph(C)*  *msr(A)*  *aph(3')-III_*  *ant(6)-Ia* | *rep20*  *rep5b*  *rep7a* |
| SE56_H6C3b | Herd 6 | Central Region of Denmark | 2020 | Pure | 2.38 | 29 | 31.97 | 183.72 | Erythromycin  (MIC >16µg/mL).  tetracycline  (MIC 32µg/mL) | *tet(K)*  *fosB_*  *mph(C)*  *msr(A)*  *ant(6)-Ia*  *aph(3')-III* | *rep20*  *rep5b*  *rep7a* |
| SE57_H6C4 | Herd 6 | Central Region of Denmark | 2020 | *Staphylococcus haemolyticus* | 2.54 | 56 | 31.96 | 111.82 | Penicillin  (MIC 0.5µg/mL).  erythromycin  (MIC >16µg/mL).  tetracycline  (MIC >32µg/mL) | *fosB*  *tet(K)*  *msr(A)*  *mph(C)*  *ant(6)-Ia*  *aph(3')-III*  *blaZ* | *rep20*  *rep7a* |
| SE58_H6C5 | Herd 6 | Central Region of Denmark | 2020 | Pure | 2.39 | 29 | 31.97 | 184.46 | Penicillin  (MIC 0.5µg/mL) | *fusB*  *blaZ*  *fosB* |  |
| SE59_H6C6 | Herd 6 | Central Region of Denmark | 2020 | *Enterococcus faecium* | 2.40 | 35 | 31.97 | 135.31 | Penicillin  (MIC 1.0µg/mL).  erythromycin  (MIC >16µg/mL).  tetracycline  (MIC 2µg/mL) | *fosB*  *mph(C)*  *msr(A)*  *ant(6)-Ia*  *aph(3')-III*  *blaZ* | *rep20* |
| SE60_H6C7 | Herd 6 | Central Region of Denmark | 2020 | Pure | 2.40 | 29 | 31.96 | 204.03 | Penicillin  (MIC 0.25µg/mL) | *fosB*  *blaZ* | *rep5b* |
