# Supplementary material for: Mapping Antimicrobial Resistance in Staphylococcus epidermidis Isolates from Subclinical Mastitis in Danish Dairy Cows
Source: Antibiotics (Basel). 2025 Jan 10;14(1):67. doi: 10.3390/antibiotics14010067 (PMC11761952; doi:10.3390/antibiotics14010067)
Supplement: Supplementary file 1 [file antibiotics-14-00067-s001.zip › Table S3.docx]

**Table S3.** Additional information regarding Cohen’s kappa statistics for evaluating the agreement between phenotypic and genotypic resistance in 60 *S. epidermidis* isolates from SCM in Danish dairy cows from 2019-2020.

**
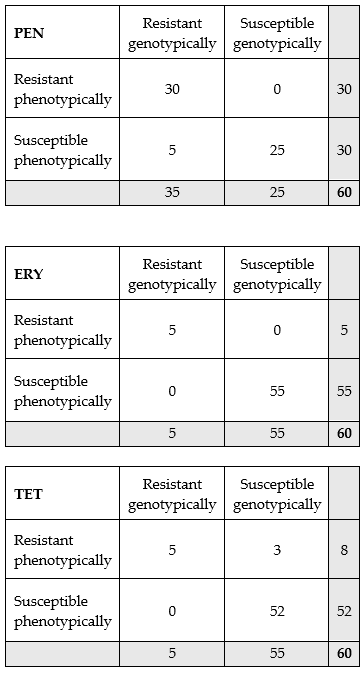
 A: B:**

| **14 antimicrobials included**  **in MIC analysis for detection of phenotypic resistance** | **ECOFF available to infer phenotypic resistance** | **n isolates phenotypic resistant** | **n isolates harboring ARG(s) corresponding to resistant phenotype** | **ARGs** |
| --- | --- | --- | --- | --- |
| FOX | Yes | 0 | 0 |  |
| CIP | Yes | 0 | 0 |  |
| ERY | Yes | 5 | 5 | *msr(A), mph(C)* |
| GEN | Yes | 0 | 0 |  |
| PEN | Yes | 30 | 35 | *blaZ* |
| TET | Yes | 8 | 5 | *tet(K)* |
| TMP | Yes | 0 | 0 |  |
| TMP+SMX | No | ND | 0 |  |
| CHL | No | ND | 0 |  |
| FFN | No | ND | 0 |  |
| SPE | No | ND |  |  |
| STR | No | ND | 9 | *ant(6)-Ia, ant(3’’)-Ia,*  *str* |
| SMX | No | ND | 0 |  |
| TIA | No | ND | 1 | *vga(A)V* |
| TMP+SMX | No | ND | 0 |  |
| **Antimicrobials not tested in MIC analysis, but ARGs detected in genomes:** |  |  |  |  |
| Fosfomycin | - | ND | 100 | *fosB* |
| Fusidic acid | - | ND | 1 | *fusB* |
| Amikacin | - | ND | 1 | *aph(3')-III* |

**A:** CHL, FFN, SPE, STR, SMX, TIA, and TMP+SMX are not reported since no ECOFFs are available to detect phenotypic resistance for these antimicrobials. FOX, CIP, GEN, and TMP are not reported, since they were fully susceptible. Fosfomycin, amikacin, and fusidic acid are not reported, although ARGs conferring resistance to these agents were detected, but these agents were not tested in MIC analysis. **B:** Demonstration of how data was ordered for PEN, ERY, and TET when calculating kappa.
